# Association between hyperuricemia and chronic kidney disease: a cross-sectional study in Bangladeshi adults

**DOI:** 10.1186/s12902-023-01304-7

**Published:** 2023-02-21

**Authors:** Zitu Barman, Mahmudul Hasan, Rakib Miah, Ananya Dutta Mou, Jaasia Momtahena Hafsa, Aporajita Das Trisha, Firoz Mahmud, Nurshad Ali

**Affiliations:** Department of Biochemistry and Molecular Biology, Shahjalal University of Science and Technology, Sylhet, 3114 Bangladesh

**Keywords:** Chronic kidney disease, Hyperuricemia, eGFR, SUA, Bangladesh

## Abstract

**Background and aims:**

Chronic kidney disease (CKD) is a public health concern worldwide and has been recognized as a significant risk factor for cardiovascular disease. The elevated level of uric acid (hyperuricemia) has been suggested to be associated with obesity, hypertension, cardiovascular disease and diabetes. However, there is limited information on the relationship between hyperuricemia and CKD. Therefore, this study aimed to estimate the prevalence of CKD and assess its relationship with hyperuricemia in Bangladeshi adults.

**Methods:**

In this study, blood samples were collected from 545 participants (398 males and 147 females) aged ≥ 18 years. Biochemical parameters such as serum uric acid (SUA), lipid profile markers, glucose, creatinine and urea were measured by colorimetric methods. The estimated glomerular filtration rate (eGFR) and CKD were determined based on serum creatinine levels with existed formula. Multivariate logistic regression analysis was performed to evaluate the association between SUA and CKD.

**Results:**

The overall prevalence of CKD was 5.9% with 6.1% in males and 5.2% in females. Hyperuricemia was prevalent in 18.7% of participants with 23.2% in males and 14.6% in females. An increasing trend of CKD prevalence was observed with increasing age in the groups. The mean eGFR level was significantly lower in male (95.1 ± 31.8 ml/min/1.73m^2^) than in female (109.3 ± 77.4 ml/min/1.73m^2^) subjects (p < 0.01). The mean level of SUA was significantly higher (p < 0.01) in participants having CKD (7.1 ± 1.9 mg/dL) than in participants without CKD (5.7 ± 1.6 mg/dL). A decreasing trend for eGFR concentration and an increasing trend for CKD prevalence was observed across the SUA quartiles (p < 0.001). In regression analysis, a significant positive association was found between hyperuricemia and CKD.

**Conclusion:**

This study showed an independent association between hyperuricemia and CKD in Bangladeshi adults. Further mechanistic studies are needed to explore the potential link between hyperuricemia and CKD.

## Background

Chronic kidney disease (CKD) is a public health concern, associated with cardiovascular disease and other complications [1]. The prevalence of CKD or kidney dysfunctions and its associated complications are increasing worldwide, especially in developing countries [2, 3].

Serum uric acid (SUA) is the final oxidation product of purine nucleotides [4]. Its overproduction and decreased excretion via kidneys are the major reasons for hyperuricemia [5]. More than 90% of all cases of hyperuricemia are the results of the impaired renal excretion of uric acid[6]. Hyperuricemia has long been recognized as a cause of gout development [7]. Recent experimental and clinical studies reported that elevated SUA is closely associated with obesity, dyslipidemia, liver dysfunction, diabetes, hypertension, metabolic syndrome and renal dysfunction [8–15]. It has also been reported that even hyperuricemia is associated with the progression of kidney disease in patients with normal kidney function [16]. The elevated level of SUA is considered an important marker for reduced renal function and likely has a causal role in the development of hypertension and cardiovascular disease [17]. In the United States, an increased prevalence of CKD was observed in 1999–2004 than in 1988–1994 [18]. Both experimental and epidemiological evidence indicates the role of SUA not only as a marker of decreased renal function and cardiovascular disease but also as a causal risk factor for CKD development and progression [19, 20]. In two large epidemiologic studies, elevated SUA has been found as an important predictor of renal disease, although, none of these studies determined its role as an independent risk factor [21, 22]. The effects of hyperuricemia on CKD development remain still an issue of active debate. Moreover, the mechanism of uric acid regulation and the association between uric acid and kidney and cardiovascular disease remain unclear [23]. Elucidating the link between SUA level and CKD would be valuable to increase our understanding of SUA-mediated kidney disease. It has been suggested that early diagnosis and treatment of modifiable risk factors for CKD are significant steps to prevent CKD progression [24]. Although some recent studies have estimated the prevalence of hyperuricemia and determined its relationship with obesity, diabetes, hypertension, metabolic syndrome, and liver dysfunction in Bangladeshi adults there is no information about the association between hyperuricemia and CKD in the country’s population. Therefore, we conducted a cross-sectional study to estimate the prevalence of CKD and its relationship with hyperuricemia in Bangladeshi adults.

## Methods

### Study area and study population

The study consisted of 545 participants (398 males and 147 females, aged ≥ 18 years). The participants were enrolled from the Sylhet region of Bangladesh including University staff, adult students and general city adults. The participants were randomly invited, we invited about two times of the participants, and however, a large number of female participants did not show interest to participate in the study due to some social and personal restrictions. Inclusion criteria: both genders and participants aged ≥ 18 years. Exclusion criteria: pregnant women, lactating mothers, individuals with a history of drug addiction and alcohol consumption and participants with anti-hyperuricemic drug intake were excluded from the study. We also excluded participants with self-reported hepatic diseases, hypothyroidism, and any infectious diseases. The ethics committee exists at the Department of Biochemistry and Molecular Biology, SUST approved all the procedures (Reference no 02/BMB/2019). Informed consent was obtained from the participants before inclusion in the study. All procedures of the study were performed in accordance with relevant guidelines and regulations.

### Anthropometric data collection

Anthropometric data including age, sex, height, weight, waist circumference (WC) and hip circumference (HC) were recorded in a structured questionnaire form following the standard procedures described elsewhere [25–31]. Height was measured to the nearest 0.1 cm using a measuring tape and weight was measured to the nearest 0.1 kg by a digital electronic LCD weighing machine (Beurer 700, Germany). Blood pressure (BP) was measured 3 times at 5 min intervals by a digital BP machine (Omron M10, Tokyo, Japan). The first BP measurement was discarded and the average value of the 2nd and 3rd measurements was considered for systolic blood pressure (SBP) and diastolic blood pressure (DBP). All the measurements were taken by trained personnel. Body mass index (BMI) was calculated as weight in kg divided by height in meters squared (kg/m^2^).

### Blood sample collection and biochemical analysis

Fasting blood samples were collected from the participants by venipuncture after overnight fasting. After collection, the blood samples were immediately placed on the icebox and transported to the laboratory. The blood samples were centrifuged by an ultracentrifuge machine (Sorvall ST 8R Centrifuge, Thermo Scientific, Germany) and serum samples were stored at − 20 °C at the Departmental laboratory until biomarker analysis. Serum level of uric acid (SUA), fasting blood glucose (FBG), serum creatinine (SCr), serum albumin (SA), serum urea (SU), serum total protein (STP), triglyceride (TG), total cholesterol (TC), low-density lipoprotein (LDL) and high-density lipoprotein (HDL) were measured by colorimetric method using commercially available kits (Human Diagnostic, Germany) with the biochemistry analyzer (Humalyzer 3000, USA) [32–34]. The accuracy of the measurements was maintained with regular standard calibration.

### Diagnostic criteria

Hyperuricemia was defined as SUA concentration *>* 7.0 mg/dL (416.4 µmol/L) in men and *>* 6.0 mg/dL (356.9 µmol/L) in women [35, 36]. An estimate of the glomerular filtration (GFR) rate was obtained by the Modification of Diet in Renal Disease (MDRD) equation: 175 × (Scr)^−1.154^ × (age)^−0.203^**×** (multiply by 0.742 if female) [18]. Kidney dysfunction or chronic kidney disease (CKD) was defined as GFR < 60 mL/min per 1.73 m^2^ and non-CKD was defined as ≥ 60 mL/min per 1.73 m^2^. CKD was also categorized as Stage 1 (normal GFR ≥ 90 ml/min), stage 2 (mild decrease in GFR 59–89 ml/min), stage 3 (moderately decrease in GFR 30–59 ml/min), stage 4 (severe decrease in GFR 15–29 ml/min and kidney failure GFR < 15 ml/min [37]. Hypertension was defined by SBP ≥ 140 mm Hg and/or, DBP ≥ 90 mm Hg and/or, intake of anti-hypertensive drugs during data collection [28, 38]. Diabetes was defined according to the American Diabetes Association 2020 as a fasting blood plasma glucose level ≥ 126 mg/dL (7 mmol/L), non-fasting plasma glucose ≥ 200 mg/dL (11.1 mmol/L) [39], or self-reported recent use of insulin or hypoglycemic drugs. Physical activity was classified as low (comfortable housework and official work), medium (walking, swimming and household stuff cleaning) and adequate/high (carrying, jogging, lifting, and/or sports). Smoking status was defined as a never-smoker and a present smoker.

### Statistical analysis

IBM SPSS statistical software (version 25.0) was used to analyse the data. SUA was divided into four quartiles by frequency test based on its concentration range: Q1 (≤ 4.7 mg/dL), Q2 (4.8–5.8 mg/dL), Q3 (5.9–6.7 mg/dL), and Q4 (> 6.7 mg/dL). An independent sample t-test (two-tailed) was used to assess the differences between gender groups and CKD and without CKD groups. One-way ANOVA was done to assess the differences in age groups and SUA quartiles. Correlations of eGFR with SUA and BUN were assessed by Pearson’s correlation coefficient test. Linearity assumption was checked between SUA and eGFR before Pearson’s correlation test. Multivariate logistic regression was applied to determine the association between hyperuricemia and CKD. Differences and correlations were indicated as significant at p < 0.05.

## Results

### Baseline characteristics of the study subjects

**Fig. 1:**
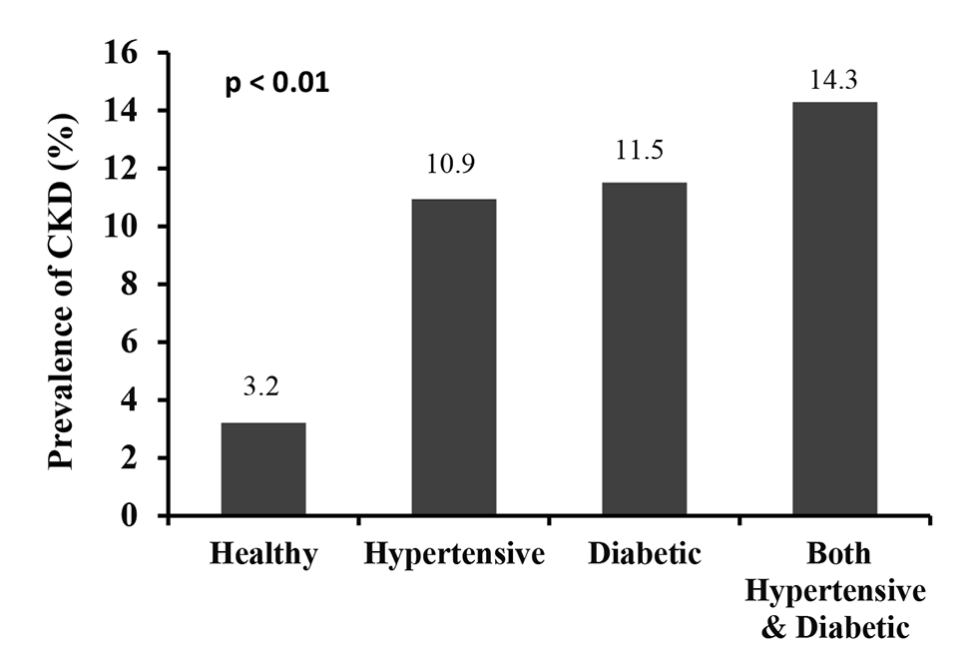
Prevalence of CKD in healthy, diabetic and hypertensive individuals. P-value was obtained from one-way ANOVA.

**Fig. 2:**
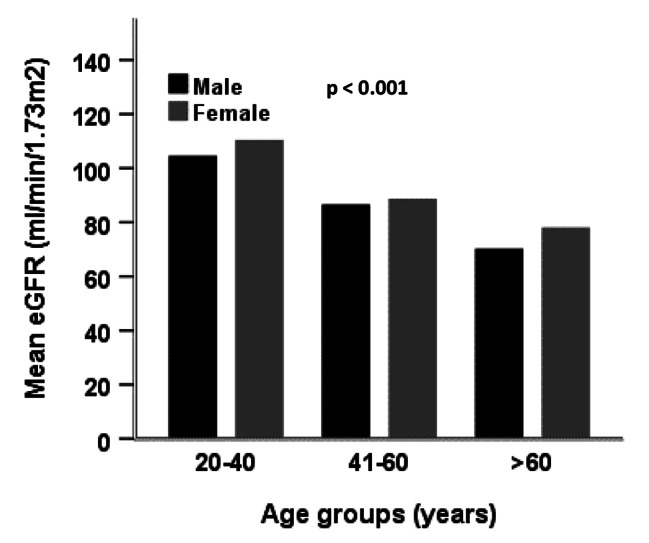
Levels of eGFR in different age groups. P-value was obtained from one-way ANOVA.

**Fig. 3:**
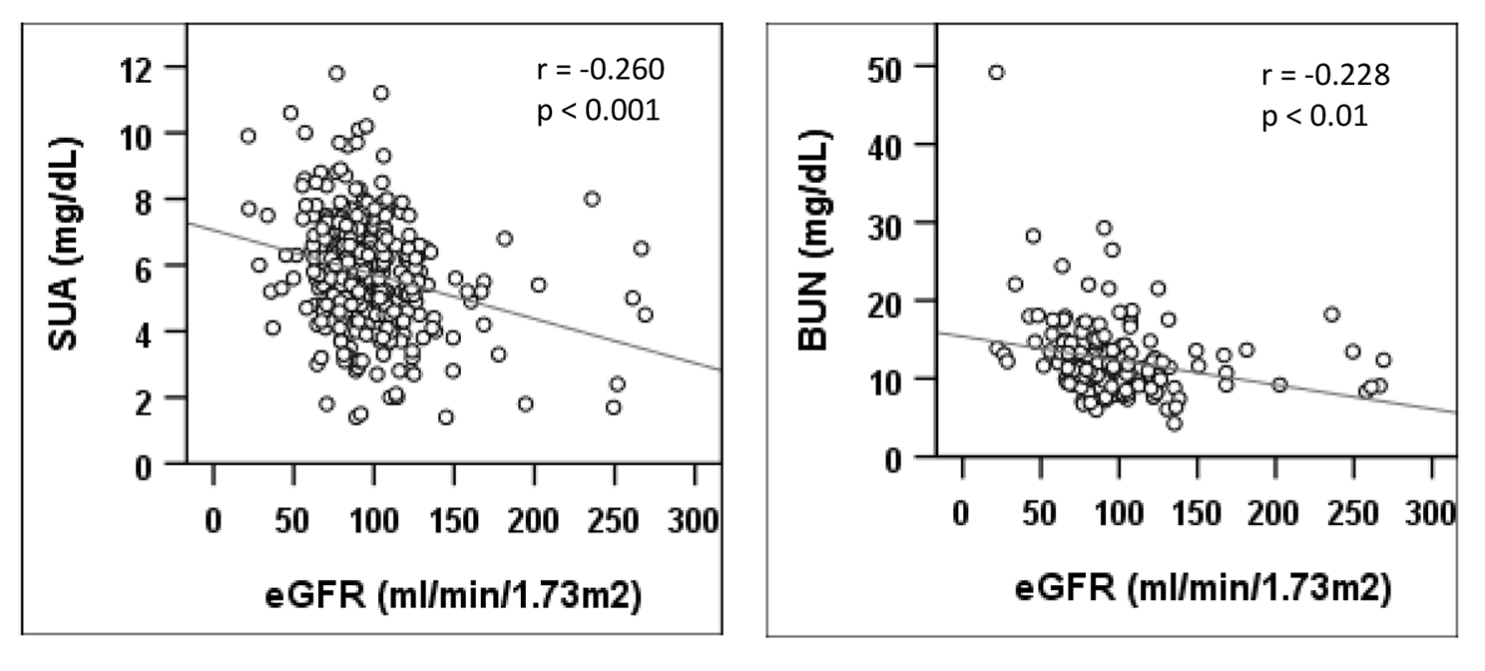
Correlations of eGFR with SUA and BUN. P-values were obtained from Pearson’s correlation coefficient test

The baseline characteristics of the study subjects are summarized in Table 1. Among 545 participants, 398 were males and 147 were females. The mean age and BMI for overall participants were 41.8 ± 13 years and 24.8 ± 3.7 kg/m^2^, respectively, with no significant differences between the gender groups. A significant difference was found for TC, HDL, LDL and SCr between the gender groups (p < 0.05 at least for all cases). The mean level of SUA for all participants was 5.8 ± 1.6 mg/dL with a significant difference between male (6.1 ± 1.8 mg/dL) and female (4.5 ± 1.8 mg/dL) participants (p < 0.01). The mean eGFR value of overall participants was 98.6 ± 47.7 ml/min/1.73m^2^ with a significant difference between males (95.1 ± 31.8 ml/min/1.73m^2^) and females (109.3 ± 77.4 ml/min/1.73m^2^) (p < 0.01). The overall prevalence of CKD was 5.9%, where male participants had a comparatively high prevalence of CKD (6.1%) than female participants (5.2%). When participants were divided into three groups, the prevalence of CKD was 3.2%, 10.9% and 11.5% in the healthy control, hypertensive and diabetic groups, respectively (Fig. 1). This prevalence was even higher in participants who were both hypertensive and diabetic (14.29%). There was an increasing trend for eGFR in the age groups (Fig. 2), which indicated the prevalence of CKD was higher in aged groups compared to younger groups. A significant negative correlation was observed for eGFR with SUA (p < 0.01) (Fig. 3), which suggested an increased prevalence of CKD with the increased level of SUA.

**Table 1:** Baseline characteristics of the study subjects by gender

Variables	Overall	Male	Female	P-value
N	545	398	147	-
Age (years)	41.8 ± 13 (85)	41.5 ± 12.9 (85)	42.5 ± 13.1 (76)	0.464
BMI (kg/m^2^)	24.8 ± 3.7 (40)	24.7 ± 3.5 (38.5)	25.1 ± 4.4 (40)	0.286
WC (cm)	85.3 ± 11.3 (165)	86.0 ± 11.4 (165)	83.6 ± 11.1 (113)	0.064
HC (cm)	91.3 ± 8.5 (114)	91.5 ± 8.3 (114)	90.7 ± 9.0 (113)	0.407
SBP (mmHg)	127.5 ± 15.5 (216)	127.6 ± 15.0 (216)	127.1 ± 17.0 (190)	0.743
DBP (mmHg)	83.4 ± 9.4 (118)	83.3 ± 9.5 (118)	83.5 ± 9.1 (112)	0.892
FBG (mg/dL)	7.3 ± 3.8 (26.9)	7.0 ± 3.6 (26.9)	8.4 ± 4.2 (26.4)	0.000
TG (mg/dL)	193.1 ± 117.8 (812.6)	195.4 ± 112.8 (726.6)	186.2 ± 132.2 (812.6)	0.466
TC (mg/dL)	206.0 ± 79.2 (584)	199.8 ± 74.5 (561.9)	224.7 ± 89.7 (584.0)	0.003
HDL (mg/dL)	33.6 ± 12.3 (112.4)	32.8 ± 12.9 (112.4)	35.9 ± 10.1 (67.8)	0.020
LDL (mg/dL)	135.4 ± 72.6 (517)	129.7 ± 65.2 (514.9)	152.3 ± 89.6 (517.2)	0.004
SUA (mg/dL)	5.8 ± 1.6 (11.8)	6.1 ± 1.8 (11.8)	4.5 ± 1.8 (9.9)	0.000
SCr (mg/dL)	0.9 ± 0.3 (2.9)	1.0 ± 0.3 (2.9)	0.7 ± 0.3 (2.3)	0.000
SA (g/L)	48.7 ± 14.4 (107.2)	48.8 ± 13.3 (107.2)	48.1 ± 17.5 (100.5)	0.726
STP (g/L)	79.9 ± 27.2 (190.1)	80.0 ± 27.5 (190.1)	79.8 ± 26.2 (167.9)	0.955
SU (mg/dL)	26.2 ± 10.3 (105.4)	26.3 ± 9.2 (62.7)	25.9 ± 13.5 (105.4)	0.821
BUN (mg/dL)	12.2 ± 4.8 (49.2)	12.3 ± 4.3 (29.3)	12.1 ± 6.3 (49.2)	0.821
eGFR (ml/min/1.73m^2^)	98.6 ± 47.7 (236)	95.1 ± 31.8 (269)	109.3 ± 77.4 (269)	0.006
Hyperuricemia (%)	18.7	23.2	14.6	0.101
CKD (%)	5.9	6.1	5.2	0.516
Smoking status (%)				0.000
Yes	22.8	29.2	0	
No	77.2	70.8	100	
Physical activity (%)				0.335
Low	21.6	20.1	26.2	
Medium	69.6	69.8	68.9	
High	8.8	10.1	4.9	

Values are presented as mean ± SD. Maximum values are indicated in the parenthesis. CKD: Chronic kidney disease, SCr: Serum Creatinine, SA: Serum Albumin, STP: Serum Total Protein, BUN: Blood Urea Nitrogen, SUA: Serum Uric Acid, SU: Serum Urea. P-values were obtained from the independent sample t-test for differences in continuous variables. Chi-square test was done to derive p-values for categorical variables. CKD was defined as eGFR < 60ml/min/1.73m^2^.

### Baseline characteristics between CKD and without control groups

The baseline characteristics of the study participants between CKD and control (participants without CKD) groups are shown in Table 2. Overall, 27 subjects had CKD according to the definition. The mean difference for SBP, FBG, SCr and SU was significant between the CKD and control groups (p < 0.05 at least for all cases). The average level of SUA was higher in the CKD group (7.1 ± 1.9 mg/dL) compared to the control 5.7 ± (1.6 mg/dL) group (p < 0.001). There was also a significant difference in BUN between the CKD and control groups (p < 0.001).

**Table 2:** Baseline characteristics of the study based on eGFR

Variables	Overall	CKD (eGFR < 60)	Normal (eGFR ≥ 60)	P-values
N	545	33	512	-
BMI (Kg/m^2^)	24.6 ± 3.6 (38.5)	23.9 ± 4.2 (32.8)	24.7 ± 3.6 (38.5)	0.247
WC (cm)	84.6 ± 11.4 (165)	85.7 ± 9.3 (104)	84.5 ± 11.6 (165)	0.629
HC (cm)	90.9 ± 8.5 (114)	90.0 ± 8.6 (109)	90.9 ± 8.5 (114)	0.593
SBP (mmHg)	127.5 ± 15.6 (216)	133.7 ± 22.7 (180)	127.1 ± 15.0 (216)	0.033
DBP (mmHg)	83.5 ± 9.6 (118)	84.0 ± 10.2 (1030	83.4 ± 9.6 (118)	0.528
FBG (mg/dL)	7.1 ± 3.6 (26.9)	9.9 ± 5.7 (26.4)	6.9 ± 3.4 (26.9)	0.000
TG (mg/dL)	192.0 ± 118 (812.6)	186.8 ± 106.1 (463.6)	192.3 ± 118.8 (812.6)	0.816
TC (mg/dL)	203.5 ± 75.6 (584)	198.4 ± 76.6 (354)	203.8 ± 75.6 (584)	0.717
HDL (mg/dL)	33.4 ± 12.1 (112.4)	32.3 ± 18.2 (67.2)	33.5 ± 11.7 (112.4)	0.614
LDL (mg/dL)	133.3 ± 69.3 (517.2)	128.7 ± 65.8 (267.9)	133.6 ± 69.6 (517.2)	0.721
SUA (mg/dL)	5.8 ± 1.6 (11.8)	7.1 ± 1.9 (10.6)	5.7 ± 1.6 (11.8)	0.000
SCr (mg/dL)	0.9 ± 0.3 (2.9)	1.7 ± 0.5 (2.9)	0.8 ± 0.2 (1.4)	0.000
SA (g/L)	49.6 ± 14.2 (107.2)	45.0 ± 12.8 (78.9)	50.0 ± 14.3 (107.2)	0.150
STP (g/L)	81.2 ± 26.5 (169.4)	69.5 ± 17.6 (113)	82.2 ± 27.0 (169.4)	0.051
SU (mg/dL)	26.2 ± 10.3 (105.4)	32.9 ± 20.5 (105.4)	25.4 ± 8.3 (62.7)	0.000
BUN (mg/dL)	12.2 ± 4.8 (49.2)	19.05 ± 10.13 (49.2)	11.9 ± 3.9 (29.3)	0.000

Values are presented as mean ± SD. Maximum values are indicated in the parenthesis. P-values were obtained from an independent sample t-test

### Baseline characteristics of the study subjects in the SUA quartiles

The participants were also characterized according to the SUA quartiles (Table 3**)**. A significant difference was found in the mean of SBP and SCr across the SUA quartiles (p < 0.05 at least for all cases). The mean level of eGFR was lower in the fourth quartile compared to other SUA quartile groups and the decreasing trend of eGFR within the quartiles was significant (p < 0.001). The prevalence of CKD was higher in the fourth quartile (9.7%) compared to the first (2.1%), second (2.7%) and third quartile (3.1%) and this prevalence difference within the quartile groups was significant (p < 0.001).

**Table 3:** Characteristics of the participants in the SUA quartiles

Variables	SUA quartiles				P-values
	Q1 (≤ 4.7)	Q2 (4.8–5.8)	Q3 (5.9–6.7)	Q4 (> 6.7)	
N	139	135	137	134	
BMI (kg/m^2^)	24.5 ± 4.0 (40)	24.5 ± 3.4 (38.4)	24.7 ± 3.5 (37.5)	25.2 ± 3.8 (38.5)	0.406
WC (cm)	85.1 ± 10.9 (113)	84.6 ± 10.0 (116)	83.5 ± 11.1 (111)	84.3 ± 9.6 (109)	0.853
HC (cm)	91.7 ± 8.3 (111)	90.7 ± 8.1 (113)	90.8 ± 8.2 (114)	90.0 ± 8.0 108.0	0.654
SBP (mmHg)	126.0 ± 14.2 (190)	127.1 ± 14.9 (180)	125.8 ± 14.1 (184)	131.7 ± 19.3 (216)	0.020
DBP (mmHg)	81.8 ± 8.0 (103)	83.9 ± 10.7 (115)	83.3 ± 9.4 (112)	84.7 ± 9.8 (118)	0.139
FBG (mg/dL)	8.7 ± 4.4 (26.9)	7.2 ± 3.7 (24.9)	6.3 ± 2.8 (18.9)	6.7 ± 3.4 (26.4)	0.000
TG (mg/dL)	171.7 ± 107.5 (675.2)	208.9 ± 147.4 (812.6)	180.5 ± 104.2 (562)	194.3 ± 87.5 (429)	0.097
TC (mg/dL)	208.5 ± 74.8 (518.6)	208.1 ± 81.9 (584)	194.0 ± 63.5 (562.8)	198.6 ± 79.7 (561.9)	0.425
HDL (mg/dL)	34.2 ± 10.7 (74.1)	34.5 ± 15.3 (112.4)	31.6 ± 9.3 (62.5)	34.1 ± 12.8 (68.7)	0.334
LDL (mg/dL)	141.3 ± 70.7 (459.7)	131.1 ± 73.5 (506.5)	129.3 ± 61.2 (517.2)	128.8 ± 74.2 (514.9)	0.567
SCr (mg/dL)	0.78 ± 0.24 (2.0)	0.85 ± 0.24 (2.0)	0.92 ± 0.25 (2.5)	1.00 ± 0.33 (2.9)	0.000
SA (g/L)	49.7 ± 18.0 (107.2)	47.1 ± 11.3 (81.6)	47.1 ± 11.5 (90.5)	49.0 ± 14.0 (92.5)	0.680
STP (g/L)	75.2 ± 26.5 (167.9)	82.8 ± 25.2 (190.1)	77.2 ± 20.2 (165.6)	86.0 ± 32.0 (169.4)	0.098
SU (mg/dL)	24.0 ± 6.0 (37.6)	26.4 ± 8.7 (52.3)	26.0 ± 10.8 (62.7)	29.3 ± 15.2 (105.4)	0.152
BUN (mg/dL)	11.3 ± 2.8 (17.5)	12.4 ± 4.0 (24.4)	12.2 ± 5.1 (29.3)	13.8 ± 7.2 (49.2)	0.155
eGFR (ml/min/1.73 m)	105.8 ± 38.0 (269.0)	97.3 ± 30.5 (261.2)	92.7 ± 25.6 (266.6)	86.6 ± 26.7 (235.9)	0.000
CKD (%)	2.1	2.7	3.1	9.7	0.000

Values are presented as mean ± SD. Maximum values are indicated in the parenthesis. P-values were obtained from one-way ANOVA. For categorical variables p-values were derived from chi-square test

### Regression analysis to evaluate the association between hyperuricemia and CKD

In regression analysis, three models were applied to assess the relationship between elevated SUA and CKD (Table 4). Model 1 was adjusted for age (years) and sex (male and female), model 2 was adjusted for model 1 and lipid profile markers (mg/dL) and model 3 was adjusted for model 2, serum albumin (g/L) and serum total protein (g/L). In all models, elevated SUA showed a significant positive association with CKD (p < 0.01 for models 1–2 and p < 0.05 for model 3).

**Table 4:** Association between hyperuricemia and CKD

	B	SE	Wald	df	OR	95% CI		P-value
						Lower	Upper	
Model 1	0.441	0.156	7.953	1	1.554	1.144	2.112	0.005
Model 2	0.493	0.162	7.344	1	1.551	1.129	2.131	0.007
Model 3	0.601	0.284	4.475	1	1.824	1.045	3.183	0.034

Multivariate logistic regression analysis was done to evaluate the association between elevated SUA and CKD. Here, CKD was the dependent variable (yes) and elevated SUA (mg/dL) was an independent variable. The reference category was normal (non-CKD). Model 1: adjusted for age (years) and gender (male and female). Model 2: model 1 + BMI, blood pressure, blood glucose and lipid profile markers (mg/dL) Model 3: model 2 + serum albumin (mg/dL) and serum total protein (mg/dL) and physical activity. OR: odds ratio, CI: confidence interval and SE: standard error

## Discussion

In the present study, we estimated the prevalence of CKD and evaluated its relationship with hyperuricemia. To the best of our knowledge, this is the first study that evaluated the association between hyperuricemia and CKD in Bangladeshi adults.

In our study, the overall prevalence of CKD was 5.9%, slightly higher in males than in females. This prevalence of CKD was higher in participants who were both diabetic and hypertensive. A high prevalence of CKD was also found in the Canadian population who were both diabetic and hypertensive [40]. In our analysis, a significant decreasing trend for eGFR was found in the age groups, which indicates that CKD prevalence is higher in aged people. Our finding is consistent with a previous study that reported a decreasing trend of eGFR, i.e., increasing prevalence of CKD in the aged population in Indonesia [41]. In the present study, the overall prevalence of hyperuricemia was 18.7% with 23.2% in males and 14.6% in females. A recent study in Bangladesh also reported a similar prevalence of hyperuricemia among Bangladeshi adults (16.6%), with 21.3% in males and 8.3% in females [9]. The prevalence of hyperuricemia has also been reported in some other Asian countries. For example, the prevalence of hyperuricemia in mainland China was 13.3% (19.4% in males and 7.9% in females) and 25.8% (34.5% in males and 11.6% in females) in Japan [19].

In this study, a significant negative association was found between eGFR and SUA among participants. We observed a decreasing trend for eGFR across the SUA quartiles. The prevalence of CKD was increased with an increased concentration of SUA in the quartile groups. Elevated SUA showed a positive and independent association with CKD. This association remained after adjustment for sex, age, serum albumin, serum total protein and lipid markers. Consistent with our findings, a previous study by Zoppini et al. reported hyperuricemia as an increased risk of CKD after adjusting for sex, age, BMI, smoking status, diabetes, blood pressure and albumin[42]. Another study by Bellomo et al. also showed a significant association between elevated SUA and CKD in multivariable analyses adjusting for several confounders [43]. Hyperuricemia has also been indicated as an independent risk factor for CKD in middle-aged and elderly adults in the Taiwanese population [44]. In an observational study in the Thai army population, the elevated level of SUA was independently associated with an increased prevalence of CKD [7]. Although there are several previous studies on this topic but most of these were conducted on a small scale and confounders were not counted in their analysis. Therefore, some recent studies mentioned above and our study, raise the possibility that hyperuricemia may mediate kidney disease and its progression.

As is known, elevated blood pressure is associated with kidney disease [45]. Our data showed that subjects with higher SUA levels had a higher mean value of SBP. There are some potential pathways through which hyperuricemia can induce hypertension such as via endothelial dysfunction, vascular smooth muscle hypertrophy, glomerular hypertrophy, and activation of the renin-angiotensin system [46–48]. However, the underlying mechanisms of SUA-mediated CKD are not well established yet. Some possible pathways may be involved in the association of hyperuricemia with CKD. For example, increasing uric acid levels can induce oxidative stress by forming urate crystals [49]. Urate crystals are very reactive compounds that may cause endothelial dysfunctions, resulting in elevated renal vascular resistance and reduced renal blood flow and GFR [49]. Vascular and endothelial dysfunctions are known to have a major role in driving CKD [50]. An elevated SUA level has also been reported to be associated with endothelial dysfunction [51]. In animal studies, increased blood pressure and tubulointerstitial injury in rats were prevented by using SUA-level lowering agents [46] which suggests an association of hyperuricemia with hypertension and renal dysfunction. Although these lines of evidence suggest an association between hyperuricemia and CKD, further mechanistic studies are needed to elucidate the potential link between them.

The major strength of this study was that data were collected from participants of a wide age range. We applied the specified method for the analysis of SUA and serum creatinine and all assays were performed in one laboratory. However, there were some limitations to our study. First, the cross-sectional nature of this study cannot determine the causal link between hyperuricemia and CKD. Second, we had no data on other kidney function tests such as haematuria, proteinuria, and imaging of the kidneys. Third, our study sample size was relatively small, thus the study cannot cover the entire population of Bangladesh. Fourth, we had no information about diuretic therapy and renin-angiotensin-aldosterone system inhibitors (RAASi) from the participants. As diuretics are one of the most important causes of secondary hyperuricemia and RAASi was found to be associated with the slow progression of CKD. Therefore, it would be valuable to extrapolate these factors to determine a more accurate association between hyperuricemia and CKD. In addition, we did not have information on the individual’s dietary habits that can affect serum creatinine levels. Despite several limitations, this study findings would be worth reference for future studies in Bangladesh.

## Conclusion

The present study showed a positive and independent association between hyperuricemia and CKD in the Bangladeshi adults and this association remained significant, even after adjusting for several confounders. National health promotion activities in Bangladesh should be increased as well as intensive screening and routine measurement of SUA may contribute to reducing the burden of hyperuricemia and associated complications in the general population in Bangladesh. Further mechanistic studies are required to explore the potential link between hyperuricemia and CKD.

## Data Availability

The datasets used and/or analysed during the current study available from the corresponding author on reasonable request.
